# The association of polymorphic markers *Arg399Gln* of *XRCC1* gene, *Arg72Pro* of *TP53* gene and *T309G* of *MDM2* gene with breast cancer in Kyrgyz females

**DOI:** 10.1186/s12885-017-3762-y

**Published:** 2017-11-13

**Authors:** Jainagul Isakova, Elnura Talaibekova, Nazira Aldasheva, Denis Vinnikov, Almaz Aldashev

**Affiliations:** 1 Institute of Molecular Biology and Medicine, 3 Togolok Moldo Str, 720040 Bishkek, Kyrgyzstan; 2 Al-Farabi Kazakh National University, School of Public Health, Al-Farabi avenue 71, 050040 Almaty, Kazakhstan

**Keywords:** Breast cancer, *XRCC1*, *TP53*, *MDM2*, Kyrgyz population

## Abstract

**Background:**

The association of genes *XRCC1, TP53* and *MDM2* with breast cancer (BC) has never been tested in Kyrgyz population. We, therefore, aimed to identify an association of alleles and genotypes of polymorphic markers *Arg399Gln* of gene *XRCC1, Arg72Pro* of gene *TP53*, and *T309G* of gene *MDM2* with the risk of BC in Kyrgyz women.

**Methods:**

This was a case-control study of 219 women of Kyrgyz origin with morphologically verified BC (*N* = 117) and 102 controls, age-matched with BC cases. The mean age of subjects in this study was 52.2 ± 10.8 years. We extracted DNA from the venous blood and genotyped polymorphic markers *Arg399Gln* of gene *XRCC1*, *Arg72Pro* of gene *TP53* and *T309G* of gene *MDM2* using polymerase chain reaction and the method of restriction fragment polymorphism.

**Results:**

Allele *399Gln* (OR 1.57; 95% CI 1.05–2.35), *Arg399Gln* of gene *XRCC1* heterozygous genotype (OR 2.77; 95% CI 1.60–4.80), the combination of *Arg399Gln/Arg72Pro* of genes *XRCC1/TP53* heterozygous genotype (OR 3.98; 95% CI 1.57–10.09), *Arg399Gln/T309G* of genes *XRCC1/MDM2* (OR 3.0; 95% CI 1.18–7.56), as well as *Arg399Gln/Arg72Pro/T309G* of genes *XRCC1/TP53/MDM2* (OR 6.40; 95% CI 1.18–34.63) were associated with BC in Kyrgyz women.

**Conclusions:**

This is the first study to identify the inter-loci interaction and to find molecular markers of individual risk of BC in Kyrgyz women.

## Background

In Kyrgyzstan, breast cancer (BC) appears to be one of the leading cancer localizations in females, remaining the second most prevalent and the third fatal type of cancer. The advanced disease is diagnosed in 40% of new cases, hampering both treatment and cure [1]. Therefore, molecular markers of predisposition to BC may be a cornerstone strategy for early detection and primary prevention of this malignant disease.

BC is known to develop from a combined effect of environmental and genetic predictors, whose interplay will determine the individual susceptibility to the negative impact of the environment. To date, series of candidate gene are under study to test the genetic component of such predisposition. Those genes regulating cellular cycle and facilitating DNA reparation as well as inducing apoptosis may have the greatest potential for that [2, 3].

XRCC1 (X-ray repair cross-complementing group) is one of the leading proteins associated with DNA reparation and coded with *XRCC1* gene of the 19th chromosome in 19q13.2 locus [4]. A polymorphic marker *Arg399Gln* is located in exon 10 of this gene and has been ben tested for an association with a few malignancies, including BC [5–7]. Moreover, BC is known to be linked with apoptosis. Protein p53 is a main driver of apoptosis after cellular genome injury, coded by *TP53* gene, located on a short arm of the 17th chromosome [8]. This gene *TP53* is known to contain polymorphic marker *Arg72Pro* in exon 4 and may play role in carcinogenesis. This marker codes arginine- and proline-containing p53 protein, differing in their capacity to activate transcription of *TP53* target genes and promote p53-mediated apoptosis [9]. MDM2 protein controls the overall amount of p53 in the cell and performs as a natural p53 inhibitor, coded by gene *MDM2*, located on a long arm of the 12th chromosome in locus 12q14.3-12q15 [10]. The first intron of *MDM2* gene contains mononucleotide polymorphism *T309G*, associated with BC in selected ethnic groups [11–13]. The association of genes *XRCC1, TP53* and *MDM2* with BC has never been tested in Kyrgyz population. We, therefore, aimed to identify an association of alleles and genotypes of polymorphic markers *Arg399Gln* of gene *XRCC1*, *Arg72Pro* of gene *TP53*, and *T309G* of gene *MDM2* with the risk of BC in Kyrgyz women.

## Methods

### Study design and patients

This was a case-control study of 219 women of Kyrgyz origin. There were 117 cases of patients with a diagnosis of BC, verified with morphological methods and treated in the inpatient department of the National Centre of Oncology in Bishkek from 2015 until 2016. The National Centre of Oncology in Bishkek approved the use of the third party data for this study. We also enrolled 102 controls with no diagnosis of BC, age-matched with BC cases. The mean age of subjects in this study was 52.2 ± 10.8 years. Almost half of both cases and controls lived in the city (Table 1). Most cases showed infiltrating ductal carcinoma, but other histological cancer types were also present. Most tumors included in this analysis were moderately differentiated. Each patient signed informed consent prior to biological material withdrawal, whereas the study followed the basic ethical principles and Declaration of Helsinki.

**Table 1:** Baseline demographic characteristics of cases and controls with histological attributes types of cancer cases

Indicator	Cases	Controls
Kyrgyz ethnicity, N (%)	117 (100)	102 (100)
Age, mean ± SD	53.8 ± 9.3	45.8 ± 8.7^a^
Urban residents, N (%)	54 (46)	62 (61)^a^
Tumor morphology		
Infiltrating ductal carcinoma	54 (46)	–
Lobular carcinoma	40 (34)	–
Less prevalent types, including solid carcinoma, medullar carcinoma, fibrosarcoma, tubular adenocarcinoma	23 (20)	–
Degree of differentiation		
Highly differentiated	5 (4)	–
Moderately differentiated	103 (88)	–
Low differentiated	9 (8)	–

*SD* standard deviation

^a^significant difference between groups using either t-test or 2*2 χ^2^ test where appropriate

### DNA extraction and genotyping analysis

Venous blood sample (5 ml) was drawn from each subject’s cubital vein for the subsequent DNA extraction. We extracted DNA from the venous blood using a conventional technique of phenol-chlorophorm extraction with subsequent DNA precipitation with 96% ethanol [14]. We genotyped polymorphic markers *Arg399Gln* of gene *XRCC1*, *Arg72Pro* of gene *TP53* and *T309G* of gene *MDM2* using polymerase chain reaction (PCR) and the method of restriction fragment polymorphism (RFP).

**Fig. 1:**
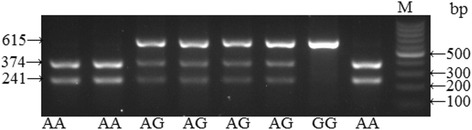
Electrophoretic separation of *Arg399Gln* polymorphic locus of *XRCC1* gene in 3% agarose gel. М – molecular scales marker with 100 bp step. *Arg/Gln* genotype are fragments 615 + 374 + 241 bp wide; *Gln/Gln* genotype 615 bp wide; *Arg/Arg* genotype 374 + 241 bp wide

We used the following primers for the amplification of *Arg399Gln* locus of gene *XRCC1*: direct 5′-TGCTTTCTCTGTGTCCA-3′, and reverse 5′-TCCAGCCTTTTCTGATA-3′. After PCR-products were restricted with MspI endonuclease, we identified alleles of *Arg399Gln* of gene *XRCC1* polymorphism via electrophoresis in 3% agarose gel. DNA fragments with the length of 615, 374, and 241 base pairs corresponded to *399Gln* allele, whereas the ones with the length of 374 and 241 base pairs were attributed to *Arg399* allele (Fig. 1) [15].

**Fig. 2:**
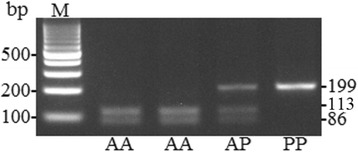
*Arg72Pro* polymorphic locus of *Р53* gene genotypes identification in 3% agarose gel after processing with BstUI endonuclease. *Pro/Pro* is 199 bp long; *Arg/Pro* is 199 + 113 + 86 bp long; *Arg/Arg* is 113 + 86 bp long. М – molecular scales marker with 100 bp step

We used the following primers for the amplification of Arg72Pro locus of gene TP53: direct 5` TTGCCGTCCCAAGCAATGGATGA – 3`, and reverse 5` TCTGGGAAGGGACAGAAGATGAC– 3`. We used BstUI endonuclease to split PCR-products after amplification. Following restriction, we obtained DNA fragments with the length of 113 and 86 base pairs, corresponding to *Arg* allele, as well as fragments with the length of 199, 113, and 86 base pairs for *Pro* allele (Fig. 2) [16].

**Fig. 3:**
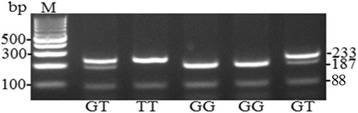
Electrophoretic separation of *T309G* polymorphic locus of *MDM2* gene. DNA fragments with the length of 233, 187, 88, 46, and 31 bp corresponding to G allele; and 233, 88, and 31 bp for T allele. М is a marker of DNA molecular mass

Finally, PCR for *MDM2 (T309G)* gene was done using the primers: direct 5`- CGGGAGTTCAGGGTAAAGGT -3`, and reverse 5` AGCAAGTCGGTGCTTACCTG-3`. In this case, MspaII endonuclease was used split PCR products. We obtained DNA fragments with the length of 233, 187, 88, 46, and 31 base pairs corresponding to G allele and 233, 88, and 31 base pairs for T allele [17] using electrophoresis (Fig. 3). Fragments 46 and 31 bp long cannot be seen because of low molecular weight.

### Statistical analysis

First, we tested the distribution of genotypes in the studied sample to fit Hardy-Weinberg equilibrium using χ2 and the critical value of *p* < 0.05 to reject the null hypothesis assuming the absence of such equilibrium. In the main analysis, we primarily report the associations of genotypes and alleles with BC, which were the primary endpoints. However, we also studied the secondary points, such as the association of genotypes and alleles with histologic types, tumor size, N or M stage and degree of differentiation. We compared the frequencies of alleles and genotypes in the groups of patients and healthy subjects using χ2 with Yates’s correction. The effect measure of the association between the allele or genotype with BC was odds ratio (OR) with the corresponding 95% confidence interval, which we tested using unadjusted regression models and, therefore, report crude OR values with their corresponding 95% CI. We ran all tests in STATISTICA 8.0. (StatSoft) and GraphPad Prism 5.0.

## Results

### Allele and genotype distribution

Table 2 shows the distribution of *Arg399Gln* of gene X*RCC1*, *Arg72Pro* of gene *TP53*, and *T309G* of gene *MDM2* in the groups of subjects with and without BC. We found that genotype frequency in the control sample corresponded to the expected one with Hardy-Weinberg principle with regard to all the tested markers.

**Table 2:** The distribution of genotypes and alleles of *Arg399Gln* of gene *XRCC1*, *Arg72Pro* of gene *TP53* and *T309G* of gene *MDM2* in BC patients of Kyrgyz ethnicity compared to healthy controls

Markers	Alleles and genotypes	Cases, n (%)	Controls, n (%)	χ^2^	р	OR	CI 95%
*Arg399Gln* gene *XRCC1* *rs25487*	Allele *Arg399*	144 (62)	146 (72)	4.46	0.034	0.64	0.42–0.95
	Allele *399Gln*	90 (38)	58 (28)			1.57	1.05–2.35
	*Arg399Arg*	38 (32)	56 (55)	13.86	0.0010	0.39	0.22–0.68
	*Arg399Gln*	68 (58)	34 (33)			2.77	1.60–4.80
	*Gln399Gln*	11 (10)	12 (12)			0.77	0.32–1.84
HWE χ2 /р		6.07/0.01	3.33/0.06				
*Arg72Pro* *gene TP53* *rs1042522*	Allele *Arg72*	164 (70)	142 (70)	0.011	0.913	1.02	0.68–1.54
	Allele *72Pro*	70 (30)	62 (30)			0.98	0.65–1.47
	*Arg72Arg*	57(49)	53 (52)	1.80	0.41	0.88	0.52–1.49
	*Arg72Pro*	50 (43)	36 (35)			1.37	0.79–2.36
	*Pro 72Pro*	10 (8)	13 (13)			0.64	0.27–1.53
HWE χ2 /р		0.04/0.83	2.80/0.09				
*T309G* gene *MDM2* *rs2279744*	Allele *T309*	120 (49)	111(46)	0.31	0.58	0.88	0.60–1.28
	Allele *309G*	114 (51)	93 (54)			1.13	0.77–1.65
	*G309G*	29 (24)	28 (27)	0.500	0.77	0.87	0.47–1.59
	*T309G*	62 (53)	55 (54)			0.96	0.56–1.64
	*T309 T*	26 (22)	19 (18)			1.24	0.64–2.42
HWE χ2 /р		0.42/0.51	0.77/0.38				

*OR* odds ratio, *CI* confidence interval, *HWE* Hardy-Weinberg equilibrium

Heterozygous genotype *Arg399Gln* and *399Gln* allele of gene *XRCC1* were associated with BC when compared to controls. This genotype *Arg399Gln* resulted in almost 3-fold increase of BC probability (OR 2.77 (95% CI 1.60–4.80)), whereas the *399Gln* allele was a marker of BC risk (OR 1.57 (95% CI 1.05–2.35)). With regard to *Arg399* allele, we found its protective effect for BC (OR 0.64 (95% CI 0.42–0.95)) (Table 1).

We failed to find similar associations of polymorphic loci *Arg72Pro* of gene *TP53* and *T309G* of gene *MDM2*, and the prevalence of these genotypes and alleles in the group of BC patients did not differ from healthy controls (р > 0.05). Therefore, taken separately, polymorphic loci *Arg72Pro* of gene *TP53* and *T309G* of gene *MDM2* were not associated with BC in the population of Kyrgyz women.

Because BC phenotype results from a combination of genotypes and alleles of various genes, rather than one gene only, making BC a genetically heterogeneous disease, we performed the analysis of intergenic (*XRCC1/TP53/MDM2*) interactions in order to identify the most meaningful gene-gene combinations, which can result in BC in Kyrgyz women.

### Gene-gene interaction between *XRCC1* and *TP53* polymorphisms

When we tested gene-gene interactions of polymorphic loci of *Arg399Gln* and *Arg72Pro*, we found statistically significant 2-loci combinations of genotypes *XRCC1/TP53* (*Arg399Gln/Arg72Pro*), which results in a significant increase of BC probability in Kyrgyz women. Thus, *Arg72Pro* heterozygous variant of gene *TP53* combined with *Arg399Gln* heterozygous genotype of gene *XRCC1* was associated with almost 4-fold increase in BC probability in the studied sample (OR 3.98 (95% CI 1.57–10.09)) (Table 3).

**Table 3:** The distribution of combinations of *Arg399Gln* of gene *XRCC1* and *Arg72Pro* of gene *TP53* polymorphic markers in Kyrgyz women with BC and controls

*XRCC1/TP53* genotypes	Cases, n (%)	Controls, n (%)	OR (95% CI)	χ^2^/р
*Arg399Arg/Arg72Arg*	19 (16)	27 (26)	Ref.	
*Arg399Arg/Arg72Pro*	18 (15)	21 (21)	1.22 (0.52–2.88)	0.20/0.65
*Arg399Arg/Pro72Pro*	1 (1)	8 (8)	0.18 (0.02–1.54)	2.97/0.085
*Arg399Gln/Arg72Arg*	32 (27)	20 (20)	2.27 (1.01–5.11)	3.23/0.07
*Arg399Gln/Arg72Pro*	28 (24)	10 (9)	3.98 (1.57–10.09)	7.58/0.0059
*Arg399Gln/Pro72Pro*	8 (7)	4 (4)	2.84 (0.75–10.81)	2.46/0.116
*Gln399Gln/Arg72Arg*	6 (5)	6 (6)	1.42 (0.40–5.09)	0.29/0.588
*Gln399Gln/Arg72Pro*	4 (3)	5 (5)	1.14 (0.27–4.80)	0.03/0.861
*Gln399Gln/Pro72Pro*	1 (1)	1 (1)	1.42 (0.08–24.18)	0.06/0.807

*OR* odds ratio, *CI* confidence interval

### Gene-gene interaction between *XRCC1* and *MDM2* polymorphisms

Compared to controls (18%), BC women had statistically significant greater prevalence of *Arg399Gln/T309G* (38%) genotype (Table 3)**.** The combination of *T309G* of gene *MDM2* heterozygous genotype with *Arg399Gln* of gene *XRCC1* heterozygous genotype was associated with a 3-fold increase of BC probability (OR 3.0 (95% CI 1.18–7.56)) (Table 4), which makes this combination of haplotypes a genetic risk factor of BC in Kyrgyz women.

**Table 4:** The distribution of combinations of *Arg399Gln* of gene *XRCC1* and *T309G* of gene *MDM2* polymorphic markers in Kyrgyz women with BC and controls

*XRCC1/MDM2* genotypes	Cases, n (%)	Controls, n (%)	OR (95% CI)	χ^2^/р
*Arg399Arg/G309G*	12 (10)	17 (17)	Reference	
*Arg399Arg/T309G*	18 (15)	31 (30)	0.82 (0.32–2.11)	0.17/0.684
*Arg399Arg/T309T*	8 (7)	8 (8)	1.42 (0.42–4.84)	0.31/0.578
*Arg399Gln/ G309G*	14 (12)	8 (8)	2.48 (0.79–7.76)	2.48/0.115
*Arg399Gln/ T309G*	38 (32)	18 (18)	3.00 (1.18–7.56)	4.49/0.034
*Arg399Gln/ T309T*	16 (14)	8 (8)	2.83 (0.92–8.73)	3.37/0.066
*Gln399Gln/ G309G*	3 (3)	3 (3)	1.42 (0.24–8.26)	0.15/0.697
*Gln399Gln/ T309G*	6 (5)	6 (5)	1.42 (0.37–5.48)	0.26/0.613
*Gln399Gln/ T309T*	2 (2)	3 (3)	1.42 (0.17–11.51)	0.11/0.74

*OR* odds ratio, *CI* confidence interval

### Gene-gene interaction between TP53 and MDM2 polymorphisms

When comparing genotype distribution of *Arg72Pro* polymorphic loci of *TP53* gene and *T309G* of *MDM2* gene, no statistical differences between BC and control groups were identified (Table 5). Of note, *Pro72Pro/G309G* genotype combination was only found in control group, but not in BC group.

**Table 5:** The distribution of combinations of *Arg72Pro* of gene *TP53* and *T309G* of gene *MDM2* polymorphic markers in Kyrgyz women with BC and controls

*TP53/MDM2* genotypes	Cases, n (%)	Controls, n (%)	OR (95% CI)	χ2/р
*Arg72Arg/G309G*	8 (7)	11(11)	Reference	
*Arg72Arg/G309T*	36 (31)	31 (30)	1.60 (0.57–4.47)	0.80/0.37
*Arg72Arg/T309T*	13 (11)	11 (11)	1.63 (0.48–5.47)	0.62/0.43
*Arg72Pro/ G309G*	21 (18)	11 (11)	2.63 (0.82–8.43)	2.69/0.10
*Arg72Pro/ G309T*	18 (15)	19 (19)	1.30 (0.43–3.98)	0.22/0.64
*Arg72Pro/ T309T*	11 (9)	6 (6)	2.52 (0.65–9.71)	1.84/0.18
*Pro72Pro/ G309G*	0 (0)	6 (6)	0.10 (0.005–2.11)	3.72/0.05
*Pro72Pro / G309T*	8 (7)	5 (5)	2.20 (0.52–9.30)	1.17/0.28
*Pro72Pro / T309T*	2 (2)	2(2)	1.38 (0.16–11.94)	0.08/0.77

*OR* odds ratio, *CI* confidence interval

### Gene-gene interaction between *XRCC1, TP53* and *MDM2* polymorphisms

We tested 27 different combinations (*XRCC1, TP53, MDM2*) and found that the interaction of *Arg399Gln/Arg72Pro/T309G* of genes *XRCC1/TP53/MDM2* heterozygous genotypes was associated with BC (χ^2^ = 5.04; р = 0.025) and increased its likelihood with an OR of 6.40 (95% CI 1.18–34.63). Additionally, we tested whether the selected polymorphic loci were associated with cancer histologic type, tumor size, N or M stage or even degree of differentiation. We found no association of these markers with any of these attributes of cancer in our patients.

## Discussion

In this case-control study, we have identified a number of genetic associations with BC in Kyrgyz women. These exposures included allele *399Gln* (OR 1.57; *p* = 0.034), *Arg399Gln* of gene *XRCC1* heterozygous genotype (OR 2.77; *p* = 0.001), as well as the combination of *Arg399Gln/Arg72Pro* of genes *XRCC1/TP53* heterozygous genotype (OR 3.98; *p* = 0.0059), *Arg399Gln/T309G* of genes *XRCC1/MDM2* (OR 3.0; p = 0.034), and *Arg399Gln/Arg72Pro/T309G* of genes *XRCC1/TP53/MDM2* (OR 6.40; *p* = 0.025).

*Arg399Gln* polymorphism of gene *XRCC1, Arg72Pro* of *TP53* gene and *T309G* of *MDM2* gene, coding enzyme synthesis with a variety of reparative and apoptosis activity, may shift the balance of reparation and injury both ways. Our findings confirm the association of heterozygous genotypes of *XRCC1/TP53/MDM2* genes with the elevated risk of BC. The strongest association of heterozygous carriage of *XRCC1/TP53/MDM2* genes with the disease calls for further analysis and more studies.

Our results highlight the role of *Arg399Gln* polymorphic locus of gene *XRCC1* in BC origin in Kyrgyz women. Our findings confirm the earlier data from Chinese [4], Polish [6], American [5], and Egyptian [7] populations, which altogether showed that *399Gln* allele and *Arg399Gln* genotype carriers had a greater BC risk compared to *Arg399* allele and *Arg399Arg* genotype. The association of *399Gln* allele and *Arg399Gln* genotype with BC may sound plausible, because published reports have shown that XRCC1 protein, having glutamine in its 399th position, has a smaller potency to repair damaged DNA, and that results in the accumulation of genetically unstable cells and may promote malignancy [2].

*Arg72Pro* of gene *TP53* polymorphic marker is located in the high proline concentration domain [8], and this domain is responsible for apoptotic functioning of р53 protein. After mutation, р53 is no more capable of activating transcription of pro-apoptotic genes, resulting in disrupted apoptosis, which altogether leads to a greater number of cells of various DNA alterations with subsequent cellular proliferation. Arginine-containing variant of р53 (*Аrg72)* protein is more potent to induce apoptosis that it’s proline-containing variant (*Pro72*) [10].

Literature data on the association of *Arg72Pro* of gene *TP53* polymorphic versions with BC are not homogenous and are somewhat contrasting. Some studies have demonstrated that *72Pro* of gene *TP53* allele has a significant association with BC [18, 19]. Other studies, in contrast, have confirmed *Arg72* allele may be more relevant [20, 21] to promote BC. Moreover, in newer studies and even meta-analyses, the associations of *Arg72Pro* of gene *TP53* marker with BC was not statistically significant [22, 23]. Such contradicting findings are likely explained by the ethnic differences in the molecular and genetic mechanisms of BC initiation and progression.

The concentration and activity of р53 cancer suppressing protein in a cell is controlled by MDM2 protein, which inactivates and accelerates degrading of р53 [10] cancer suppressing protein, thus, hampering DNA reparation and, therefore, promotes, carcinogenesis.

With regard to *T309G* of gene *MDM2* polymorphic marker, we failed to demonstrate its statistically significant association with BC in a group of BC females compared to controls**.** However, *Arg72Pro* heterozygous variant in combination with *Arg399Gln* of gene *XRCC1* heterozygous genotype was associated with a 4-fold increase in the probability of BC (OR 3.98 (95% CI 1.57–10.09)).

A combination of risk genotypes of a number of candidate genes, producing additive effect, may result in simultaneous DNA reparation disorder and apoptosis, leaving some potential for a new phenotype formation [11].

The latest meta-analysis [24] of 19 publications with a total of 9788 BC cases and 11,195 controls has shown that *T309G* of gene *MDM2* polymorphic locus is associated with BC both in Asian and Caucasian populations. The magnitude of such association was most pronounced when *T309G* of gene *MDM2* heterozygous genotype was present with the greatest effect in Asians (OR 1.21 (95% CI 1.03–1.41)); *p* = 0.02), compared to Caucasians (OR 1.09 (95% CI 1.00–1.18); *p* = 0.04). Of note, GG genotype of polymorphic locus of *MDM2* gene is considered a risk factor for BC in Taiwanese women (OR 3.05 (95% CI 1.04–8.95)); p = 0.04) [12].

Therefore, combined with the findings of other cohorts, our data confirmed the individual susceptibility to BC resulting from polymorphic markers of DNA repair genes *(XRCC1)*, apoptosis genes *(TP53)*, as well as of apoptosis inhibition genes *(MDM2)*.

## Conclusions

Our study has enabled to identify the inter-loci interaction and to find molecular markers of individual risk of BC in Kyrgyz women. The list of potential risk factors for BC in Kyrgyz females may include *399Gln* allele and *Arg399Gln* of gene *XRCC1* heterozygous genotype, as well a combination of heterozygous genotypes of *Arg399Gln/Arg72Pro* of genes *XRCC1/TP53, Arg399Gln/T309G* of genes *XRCC1/MDM2* and *Arg399Gln/Arg72Pro/T309G of genes XRCC1/TP53/MDM2.*

Identification of risk combinations of genes *XRCC1*, *TP53* and *MDM2* with BC may increase the study validity and determine groups of women with high individual risk of BC, which may help in the prevention, early detection and effective cure of this condition.

## Abbreviations

BC: breast cancer
CI: confidence interval
MDM2: mouse double minute 2
OR: odds ratio
XRCC1: X-ray repair cross-complementing group
ТР53: tumor protein
